# Advances in Male Reproductive Toxicology of Nanoplastics: Potential Risks to Human Reproduction—A Systematic Review

**DOI:** 10.3390/ijms27073191

**Published:** 2026-03-31

**Authors:** Hang Han, Bo Xu, Xiuying Pei, Xufeng Fu

**Affiliations:** Key Laboratory of Fertility Preservation and Maintenance of Ministry of Education, School of Basic Medical Sciences, Ningxia Medical University, Yinchuan 750004, China; hanhang2025@nxmu.edu.cn (H.H.);

**Keywords:** micro/nanoplastics, environmental toxicology, reproductive health, reproductive toxicology, environmental pollution, health risk

## Abstract

Microplastics and nanoplastics (MPs/NPs) have emerged as pervasive and persistent environmental contaminants, prompting significant concerns about their potential risks to human health. This review provides a comprehensive synthesis of the current state of knowledge on the reproductive toxicity induced by MPs/NPs, with a particular focus on nanoplastics (NPs, <100 nm) due to their enhanced ability to cross biological barriers and induce cellular damage. Following a systematic literature search, we detail the multiple exposure pathways—including ingestion, inhalation, and dermal contact—through which MPs/NPs enter the human body and are disseminated to reproductive tissues. The core of this review elucidates the fundamental mechanisms underlying MPs/NPs-induced reproductive damage. Compelling evidence from in vitro, animal, and initial human studies demonstrates that MP/NP exposure can lead to diminished sperm quality and motility, testicular histological disruption, impaired ovarian folliculogenesis, granulosa cell apoptosis, and dysregulation of key reproductive hormones. We further summarize potential therapeutic interventions, such as antioxidants and traditional Chinese medicine compounds, and discuss key preventive and regulatory strategies. Despite the advancing evidence, critical challenges remain, including quantifying actual human exposure levels, understanding the effects of chronic, low-dose exposure, and elucidating the combined toxicity of MPs/NPs with other environmental pollutants. This comprehensive analysis underscores the urgent need for further mechanistic research, robust epidemiological studies, and the formulation of evidence-based public health policies to mitigate exposure and safeguard global reproductive health.

## 1. Introduction

In recent years, due to their widespread use, micro/nanoplastics (MPs/NPs) have emerged as a significant class of environmental pollutants. Their potential harm to human health is currently a subject of intense research. Microplastics (MPs, <5 mm in diameter) and nanoplastics (NPs, 1–100 nm in diameter) are small plastic particles formed by the mechanical, biological, photodegradation, and photooxidative degradation of various plastic products [1]. Plastic products are advantageous in daily life due to their low cost and convenience. Furthermore, plastics have broad applications across numerous fields, including food packaging, toys, clothing, medical equipment, building materials, and cosmetics [2]. Although plastic products have brought convenience to modern life, their widespread use and improper disposal have increased pollution and human health risks in recent decades. As of 2015, more than 79% of plastic waste was disposed of in landfills or released into the natural environment. According to current trends in plastic production and disposal, approximately 12,000 million tons of plastic waste will accumulate in the environment by 2050 [3]. Even if restrictions on plastic production and consumption are implemented now, it is estimated that 710 million tons of plastic waste will still be present in marine and terrestrial systems worldwide by 2040 [3].

A growing number of studies have demonstrated that oxidative stress is a critical factor in male infertility. Up to 80% of infertile men exhibit significantly elevated levels of reactive oxygen species (ROS) in their sperm, a condition termed “male oxidative stress infertility.” Oxidative stress plays a key role in male infertility by increasing intracellular ROS and disrupting the antioxidant system during spermatogenesis, and it is recognized as one of its primary causes [4]. It is now widely recognized that common environmental endocrine disruptors, such as bisphenol A and phthalate esters, induce male reproductive disorders by elevating oxidative stress levels. Metabolomics studies have shown that the decline in human semen quality in China is closely associated with metabolites of phthalate esters and perfluoroalkyl substances, suggesting that oxidative stress induced by environmental factors is a significant contributor to this decline [5].

This article reviews the toxicological research of MPs/NPs and the current situation of reproductive health issues, with emphasis on NPs due to their unique physicochemical properties and greater biological reactivity. We aim to provide a comprehensive synthesis of mechanisms, evidence, and future directions for environmental protection and reproductive health research.

## 2. Methods

**Figure 1:**
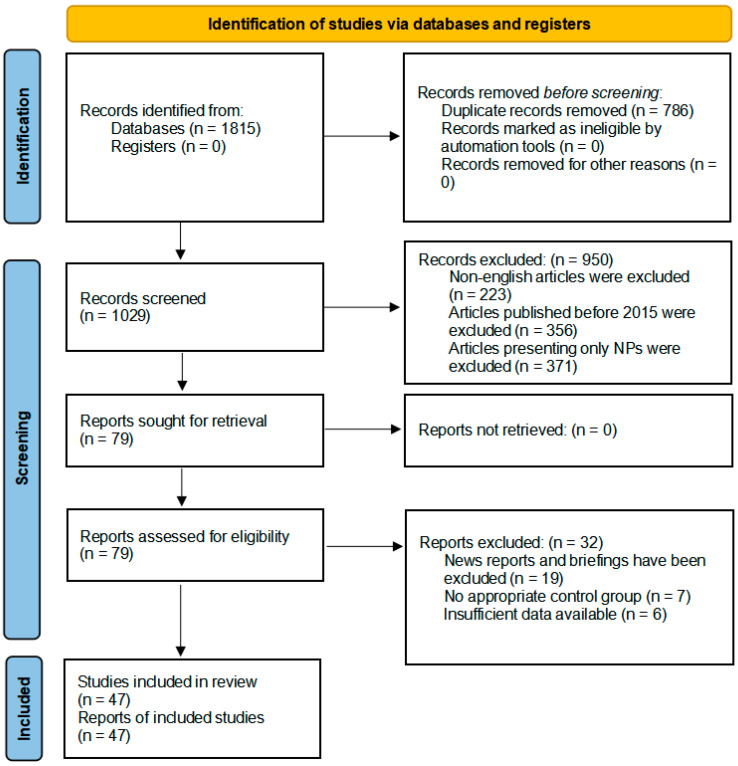
Selection criteria and process.

Literature Screening Process: The screening and reporting of the literature followed the Preferred Reporting Items for Systematic Reviews and Meta-Analyses (PRISMA) 2020 statement. The PRISMA flow diagram (Figure 1), prepared in accordance with the PRISMA 2020 statement, provides a detailed summary of the study identification, screening, and selection process. All the figures and tables in the text were created using the Microsoft Office 2021 software.

Inclusion Criteria: A literature search was conducted using the following peer-reviewed scientific databases: Web of Science (London, UK), Elsevier (Amsterdam, The Netherlands), and PubMed (Bethesda, MD, USA). The search employed the following keywords: “micro/nanoplastics,” “environmental toxicology,” “reproductive health,” “reproductive toxicology,” “environmental pollution,” and “health risk.” Only studies published between 2015 and 2025 were considered.

Exclusion Criteria: Non-English articles were excluded. Articles published before 2015 were excluded. Studies that focused exclusively on nanoplastic materials, without relevance to reproductive toxicity, were excluded.

Outcome Measures: The assessment of nanoplastic-induced reproductive toxicity primarily focused on three key outcomes: (1) total sperm count and motility; (2) changes in androgen levels; and (3) histopathological alterations in testicular tissue.

A total of 1815 records were identified through database searching. After removing 786 duplicates, 1029 records remained for title and abstract screening. Of these, 950 records were excluded (223 non-English articles, 356 articles published before 2015, and 371 articles presenting only NPs), leaving 79 full-text articles for eligibility assessment. Subsequently, 26 full-text articles were excluded (19 news reports/briefings, 7 without appropriate control groups, and 6 with insufficient data), resulting in 47 studies that were included in the quantitative synthesis.

## 3. Results and Discussion

### 3.1. Potential Environmental Hazard of Micro/Nanoplastics

In addition to conventional plastic products and waste, discarded disposable masks have emerged as a significant source of MPs/NPs since the outbreak of the COVID-19 pandemic. Disposable medical or surgical masks are primarily manufactured from various plastic materials, including polypropylene, polyurethane, polyacrylonitrile, polystyrene, polycarbonate, and polyethylene. The global monthly usage of disposable masks exceeded 129 billion in 2020 [6]. Consequently, a large volume of waste surgical masks has entered the environment, where they can degrade into harmful MPs/NPs [7]. Studies have demonstrated that waste medical masks release substantial quantities of MPs/NPs into rivers, lakes, and oceans following long-term natural weathering and photodegradation [8,9].

MPs/NPs can readily enter the human body through ingestion, inhalation, or dermal contact, subsequently accumulating in tissues and organs, where they may adversely affect organismal behavior and metabolism (Table 1) [10]. Recently, MPs/NPs have been detected in various matrices, including tea bags, bottled water, human fecal samples, human blood [11], and even human placental tissue [12]. Thus, MPs/NPs pose a serious threat to organisms and present immeasurable potential risks to the entire ecosystem [13].

However, accurately quantifying environmental levels of MPs/NPs remains challenging due to current technological limitations and an incomplete understanding of the processes and rates governing the degradation of MPs into NPs. Consequently, MPs/NPs are considered among the most hazardous environmental pollutants.

### 3.2. Potential Health Risks of MPs/NPs

Ingested or absorbed MPs/NPs can accumulate in various organisms and be transferred to higher trophic levels through the food chain, potentially inducing acute toxic effects at both the cellular and molecular levels [14]. For example, polystyrene nanoplastics (PS-NPs) taken up by algae and zooplankton can be transmitted through aquatic food chains, subsequently disrupting lipid metabolism and locomotor behavior in crucian carp. Studies have shown that charged PS-NPs exhibit greater toxicity than their uncharged counterparts [15,16]. Furthermore, MPs/NPs can adversely affect the growth, development, and reproduction of organisms [17]. Moreover, MPs/NPs readily combine with other environmental pollutants, such as heavy metals and bisphenol A (BPA), which can enhance their overall toxicity to organisms [18]. These findings underscore that the combined toxic effects of MPs/NPs and other environmental pollutants on human health warrant further investigation.

**Figure 2:**
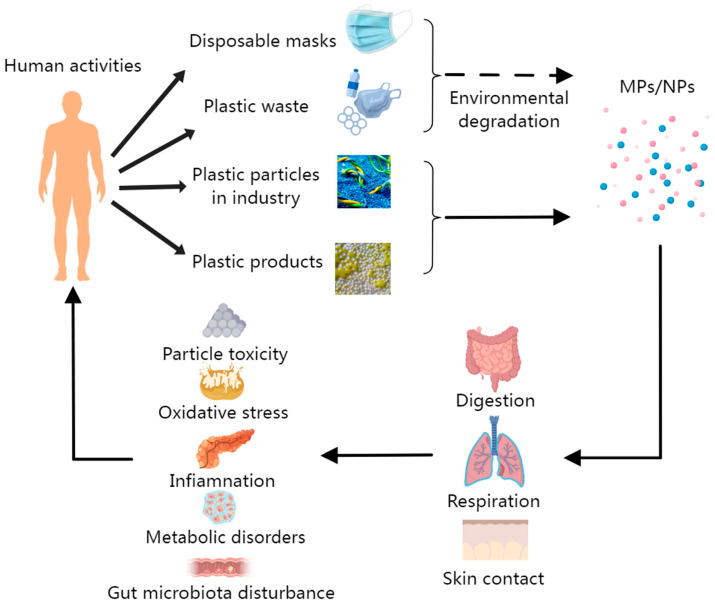
Potential MPs/NPs risks to human health.

MPs/NPs are known to enter the circulatory system through ingestion, inhalation, and dermal absorption [19] (Figure 2). Recent studies have detected MPs/NPs in several human tissues; however, their specific risks to human health remain unclear. In parallel, other studies have characterized the toxicity of MPs/NPs in various human cell types. In the human monocyte lymphoma cell line THP-1, the cytotoxic effects of MPs/NPs were found to depend primarily on particle diameter, dose, and exposure duration [20]. It has been suggested that particles with a diameter of less than 10 nm may enter cells through mechanisms analogous to gas diffusion. An analysis of the correlation between particle diameter and toxicity revealed that 64 nm PS-NPs induced greater toxicity in human acute monocytic leukemia cells (Monomac-6) compared to 202 nm and 535 nm particles. Furthermore, exposure to 44 nm NPs resulted in significantly higher toxicity—including increased lethality and proinflammatory cytokine production—in the human gastric adenocarcinoma cell line AGS compared to exposure to 100 nm NPs [16].

Conversely, MPs/NPs larger than 150 μm may induce local inflammation in the intestinal tract, thereby increasing intestinal permeability and facilitating the translocation of smaller MPs/NPs (<150 μm) into the systemic circulation, where they can exert toxic effects on distant tissues and organs. These studies collectively indicate that exposure to MPs/NPs can be directly toxic to various human cell types and may facilitate the infiltration of these particles into tissues and organs. Consequently, researchers have increasingly focused on the potential health effects of MPs/NPs. In summary, the toxicity of MPs/NPs to organisms appears to increase with decreasing particle size. Therefore, this review will focus specifically on NPs in the subsequent sections.

Human activities (including disposable mask waste, general plastic waste, industrial plastic particle emissions, and plastic product use) are the primary sources of MPs/NPs in the environment. These plastic particles can enter the human body through three main exposure routes: digestion (oral ingestion), respiration (inhalation), and skin contact. Once internalized, MPs/NPs can induce a cascade of adverse biological effects, including particle toxicity, oxidative stress, inflammation, metabolic disorders, and gut microbiota disturbance, ultimately posing risks to human health.

### 3.3. Fundamental Theories of MPs/NPs-Induced Reproductive Damage

#### 3.3.1. Biological Response Mechanisms of the Reproductive System to MP/NP Exposure

Upon exposure to MPs/NPs, the reproductive system mounts a series of biological responses. The entry of MPs/NPs into reproductive tissues can trigger an oxidative stress response, characterized by elevated intracellular levels of ROS. This oxidative stress can subsequently damage key biological macromolecules, including lipids, proteins, and DNA [21]. Studies have demonstrated that MP/NP exposure can significantly elevate ROS levels in reproductive cells, leading to lipid peroxidation, compromised cell membrane integrity, and impaired cellular function [22].

Concurrently, MPs/NPs may induce inflammatory responses by activating relevant signaling pathways, leading to the release of pro-inflammatory cytokines and subsequent inflammatory damage in reproductive tissues [23]. Several studies have reported upregulation of inflammation-related gene expression and increased inflammatory cell infiltration in reproductive organs following MP/NP exposure, confirming that inflammation plays a critical role in MPs/NPs-induced reproductive toxicity [24]. Additionally, MPs/NPs may interfere with metabolic processes within the reproductive system, disrupting energy homeostasis and hormone synthesis, thereby indirectly impairing reproductive function. For example, MPs/NPs may interfere with steroid hormone synthesis, disrupt endocrine balance within the reproductive axis, and consequently impair the development and maturation of germ cells [25].

#### 3.3.2. Mechanisms of MPs/NPs Toxicity on Reproductive Cells

MPs/NPs exert significant toxic effects on reproductive cells through multiple interrelated mechanisms. At the cellular level, MPs/NPs can directly damage the plasma membrane and intracellular organelles of reproductive cells, thereby disrupting their normal physiological functions. Research has demonstrated that MP/NP exposure can induce structural damage to mitochondria in sperm cells, compromising energy supply and subsequently reducing sperm motility and progressive movement [24].

**Figure 3:**
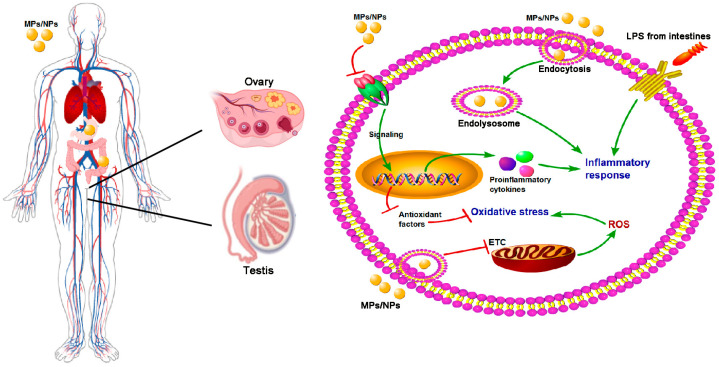
Potential toxicological mechanisms of MPs/NPs.

At the molecular level, MPs/NPs can interfere with gene expression profiles and disrupt signal transduction pathways in reproductive cells. Transcriptomic analyses of reproductive cells exposed to MPs/NPs have revealed significant alterations in the expression of numerous genes involved in germ cell development, differentiation, and function [26]. For instance, key genes governing spermatogenesis and oocyte maturation may be downregulated, thereby compromising normal germ cell development. Furthermore, MPs/NPs can induce cell cycle arrest in reproductive cells by dysregulating key cell cycle checkpoints, thereby inhibiting cell proliferation and differentiation [22]. Simultaneously, direct interactions between MPs/NPs and intracellular biomolecules—including proteins and nucleic acids—can lead to protein dysfunction, DNA damage, and ultimately compromise the functional integrity and genetic stability of reproductive cells [21] (Figure 3).

MPs/NPs can accumulate in the reproductive system following systemic circulation and exert adverse effects on reproductive function through multiple cellular pathways. These particles enter cells via endocytosis, triggering endolysosomal dysfunction and subsequent release of pro-inflammatory cytokines, which drives inflammatory responses. Concurrently, MPs/NPs disrupt the mitochondrial electron transport chain (ETC), leading to excessive reactive oxygen species (ROS) production and oxidative stress, which further impairs antioxidant defense systems. Additionally, MPs/NPs may directly or indirectly modulate nuclear signaling pathways, altering gene expression, and can synergize with intestinal lipopolysaccharide (LPS) to exacerbate inflammatory and oxidative stress cascades, ultimately contributing to reproductive dysfunction.

### 3.4. Epidemiological Studies on MPs/NPs-Induced Reproductive Damage

#### 3.4.1. Epidemiological Investigations on MP/NP Exposure and Reproductive Health

A growing body of research has investigated the potential association between MP/NP exposure and adverse reproductive health outcomes. In vivo studies utilizing mouse models have demonstrated that oral administration of varying doses of polystyrene MPs/NPs (PS-NPs) induces reproductive toxic effects, including reduced sperm quality and impaired testicular architecture. For example, one study reported that after 35 days of oral exposure to PS-NPs, mice in the medium- and high-dose groups exhibited decreased sperm quality and structural damage to the testes, with effects showing a positive dose–response relationship [27].

In human studies, while direct evidence remains limited, researchers have detected the presence of microplastics and nanoplastics in reproductive tissues such as the placenta, suggesting that human reproductive health may be at risk from MP/NP exposure [28]. Additionally, several epidemiological surveys have attempted to assess the relationship between MP/NP exposure levels and reproductive health indicators in human populations using questionnaires and biological sample analysis. Although a direct causal relationship has not yet been firmly established, these studies provide important foundational evidence for further investigating the impact of MPs/NPs on human reproductive health.

#### 3.4.2. Assessment of Reproductive Risks from MP/NP Exposure in Different Populations

Exposure levels and associated reproductive risks may vary considerably across different populations due to differences in living environments, occupational settings, and other factors. Occupational groups involved in plastic production and processing may experience higher levels of exposure to MPs/NPs through inhalation and dermal contact, potentially placing them at increased reproductive risk [29]. Studies monitoring reproductive health indicators in these occupational cohorts have reported alterations in parameters such as sperm quality and reproductive hormone levels compared to non-occupationally exposed populations. However, owing to the presence of numerous confounding factors, accurately assessing the independent effect of MP/NP exposure on reproductive health remains challenging.

For the general population, exposure occurs primarily through dietary intake and inhalation of airborne particles. Studies have identified seafood, drinking water, and other dietary components as potential sources of MPs/NPs. For instance, detectable levels of MPs/NPs have been reported in various seafood products, suggesting that long-term consumption may contribute to cumulative human exposure. Analyses of exposure assessments and reproductive health data in general populations have suggested potential associations between adverse reproductive outcomes and MP/NP exposure in certain regions; however, larger-scale, long-term prospective studies are required to confirm these findings [30].

#### 3.4.3. Analysis of Environmental Exposure Pathways for MPs/NPs and Reproductive Damage

MPs/NPs are ubiquitous environmental contaminants, and human exposure can occur through multiple pathways. Dietary intake represents a major exposure route, as MPs/NPs in food can originate from contaminated water sources, soil, and plastic packaging materials. Research has detected MPs/NPs in drinking water and various food products. For example, bottled water has been shown to contain measurable levels of MPs/NPs particles, suggesting that long-term consumption may increase overall exposure risk [31]. Inhalation represents another significant route of exposure. In industrial and urban environments, airborne MPs/NPs can be readily inhaled. Dermal contact may also contribute to exposure, particularly through the use of cosmetics or the handling of plastic products containing MPs/NPs. Furthermore, plastic medical devices used in healthcare procedures may release MPs/NPs, potentially increasing patient exposure during medical interventions. These diverse exposure pathways may affect the reproductive system to varying degrees, underscoring the need for further research to elucidate their specific contributions to reproductive toxicity [32].

However, detection does not equate to toxicity. Epidemiological studies attempting to establish causal links between NP exposure and adverse reproductive outcomes face several significant challenges:

Lack of quantitative exposure assessment: Current analytical methods are unable to accurately measure individual internal doses of NPs.

Confounding factors: Co-exposure to other environmental pollutants (e.g., phthalates, BPA, heavy metals), lifestyle factors, and genetic variability complicate causal inference.

Small sample sizes: Most studies to date are exploratory in nature and possess limited statistical power.

Cross-sectional design: The cross-sectional design of most studies precludes the establishment of temporal relationships between exposure and outcome.

Preliminary studies in occupationally exposed populations have suggested alterations in sperm parameters and hormone levels; however, confounding by co-exposures and limited sample sizes preclude definitive conclusions. Large-scale, longitudinal cohort studies employing improved exposure assessment techniques are urgently needed.

### 3.5. Ferroptosis, Metabolic Disruption, and Gut Microbiota Dysbiosis: Key Mechanisms of Nanoplastic Reproductive Toxicity

In recent years, substantial progress has been made in elucidating the toxic effects of NPs on the male reproductive system and the underlying mechanisms driving these effects. A growing number of studies have identified key molecular pathways through which NPs induce reproductive injury, offering diverse mechanistic perspectives. Research has demonstrated that NPs can accumulate in testicular tissue, disrupt the blood-testis barrier, impair spermatogenesis, and induce damage in spermatogenic cells. Utilizing RNA-seq analysis coupled with experimental validation, a seminal study reported for the first time that NPs induce ferroptosis in spermatogenic cells. This study further identified nuclear factor erythroid 2-related factor 2 (Nrf2) as a key regulatory factor that plays a protective role in this process, thereby providing novel insights into the mechanisms of nanoplastic reproductive toxicity [33].

Subsequent investigations employing the mouse spermatocyte cell line GC-2spd(ts) integrated transcriptomic and metabolomic approaches to systematically characterize the effects of exposure to 50 nm and 90 nm polystyrene nanoplastics (PS-NPs) on cell proliferation, viability, and associated molecular networks. The results demonstrated that PS-NP exposure interfered with cell cycle regulation, autophagy, ferroptosis, and redox homeostasis, while also significantly disrupting key metabolic pathways—including amino acid metabolism, cyanoamino acid metabolism, and purine/pyrimidine metabolism. These findings suggest that metabolic reprogramming is a critical component of NPs-induced reproductive toxicity [34].

Furthermore, in vivo studies revealed that exposure to 50 nm and 90 nm PS-NPs for 30 days significantly reduced sperm count and motility in mice, increased the rate of sperm abnormalities, and altered testosterone levels. Concurrently, PS-NP exposure disrupted the structure and composition of the gut microbiota and altered the fecal metabolite profile, with several common differential metabolites—including 4-deoxyerythronic acid, 8-iso-15-keto-PGE2, and sphingosine—being identified. These findings suggest that PS-NPs may interfere with male reproductive function through modulation of the “gut microbiota-metabolite” axis, thereby identifying potential biomarkers for health risk assessment of nanoplastic exposure [35].

Most recently, a study has further elucidated the upstream molecular mechanism by which NPs induce ferroptosis in spermatocytes. Specifically, NPs were shown to promote the degradation of ferroportin 1 (FPN1), a key iron exporter, by regulating its ubiquitination. This leads to intracellular iron overload and subsequent lipid peroxidation, ultimately triggering ferroptosis and mediating male reproductive injury [36].

In summary, these studies collectively elucidate the complex, multifactorial mechanisms through which NPs mediate male reproductive injury, including the induction of ferroptosis, disruption of metabolic homeostasis, and modulation of the gut microbiota-metabolite axis. These findings provide novel perspectives on the reproductive toxicity of nanoplastics and identify potential intervention targets for the prevention and treatment of associated male reproductive disorders (Table 2).

### 3.6. Therapeutic Strategies for Nanoplastic-Induced Reproductive Injury

#### 3.6.1. Pharmacological Interventions for Reproductive Injury Following Nanoplastic Exposure

Pharmacological intervention represents a promising therapeutic strategy for mitigating reproductive injury induced by nanoplastic exposure. Antioxidant compounds have been investigated for their capacity to attenuate NPs-induced oxidative stress and subsequent cellular damage. For example, in vitro experiments have demonstrated that the hydrogen sulfide (H_2_S) donor NaHS can alleviate polystyrene NP-induced mitochondrial apoptosis and excessive autophagy in mouse spermatocytes through modulation of the Nrf2 and PGC-1α signaling pathways, thereby exerting protective effects on reproductive cells [37].

Additionally, certain bioactive compounds derived from traditional Chinese medicine have demonstrated potential therapeutic benefits in preclinical models. Paeoniflorin, for instance, was reported to ameliorate the polystyrene nanoplastic-induced decline in reproductive capacity and reduce germ cell apoptosis in Caenorhabditis elegans through inhibition of the DNA damage checkpoint [38]. In vivo experiments confirmed that paeoniflorin treatment improved reproductive capacity and attenuated germ cell apoptosis in the nematode model. These findings offer novel insights and promising directions for the pharmacological management of NPs-induced reproductive injury; however, further clinical trials are essential to validate their efficacy and safety in humans.

#### 3.6.2. Preventive Measures for Nanoplastic-Associated Reproductive Injury

Preventing NPs-associated reproductive injury is of paramount importance. Key preventive strategies include reducing the generation and environmental release of NPs at the source. Strengthening regulations governing plastic production and use, promoting the adoption of biodegradable alternatives, and reducing consumption of single-use plastic products can collectively help mitigate the environmental burden of NPs [39].

At the individual level, adopting protective measures can significantly reduce personal nanoplastic exposure. Such measures include opting for glass, ceramic, or other non-plastic containers over plastic ones; limiting consumption of seafood that may contain NPs; and consuming filtered water to minimize intake. Furthermore, enhancing public awareness of the potential hazards associated with NPs and promoting environmental consciousness can contribute to reduced exposure at the population level. Concurrently, occupational safety measures—such as the use of protective masks and gloves—should be reinforced for occupationally exposed populations to minimize inhalation and dermal contact with NPs [40].

#### 3.6.3. Rehabilitation Approaches for Nanoplastic-Induced Reproductive Injury

Therapeutic approaches for individuals who have already sustained NPs-induced reproductive injury are currently under investigation. Emerging evidence suggests that modulation of the gut microbiota may represent a viable strategy for ameliorating reproductive damage following nanoplastic exposure. For instance, probiotic supplementation has been shown to help restore gut microbiota balance, alleviate NPs-induced intestinal inflammation and oxidative stress, and consequently exert indirect protective effects on reproductive function [41].

Furthermore, for oxidative damage induced by NPs, antioxidant administration may aid in restoring reproductive cell function. In preclinical animal studies, antioxidant treatment has been shown to reduce oxidative damage in reproductive cells and improve sperm quality and motility. Additionally, in cases of hormone imbalance, interventions such as hormone replacement therapy may be considered to restore normal reproductive function. However, these therapeutic strategies remain largely at the preclinical stage, and further clinical studies are warranted to verify their efficacy and safety in humans.

### 3.7. Controversies and Future Perspectives in MPs/NPs and Reproductive Injury Research

#### 3.7.1. Contentious Issues in NPs Reproductive Toxicity Research

Several contentious issues persist within the field of nanoplastic reproductive toxicity research. First, there is considerable variability in the toxic effects of NPs reported across different studies. While some studies demonstrate that NPs can induce significant reproductive toxicity, including impaired sperm quality and ovarian dysfunction, other investigations have reported no substantial reproductive damage under specific exposure conditions [42]. These discrepancies are likely attributable to variations in multiple experimental parameters, including particle type, size, concentration, and exposure duration, as well as the specific animal models employed [42]. For example, particles composed of different polymers possess distinct surface properties and chemical compositions, which may lead to divergent interactions with reproductive tissues and, consequently, variable toxicological outcomes [43].

Second, the precise molecular mechanisms underlying the impact of NPs on reproductive function remain incompletely elucidated. Although oxidative stress, inflammatory responses, and endocrine disruption have been proposed as key mechanisms, the complex interplay between these pathways and their relative contributions across different species and tissue types warrants further investigation. Third, the combined effects of NPs with other environmental contaminants on reproductive health remain a subject of considerable debate. Their joint toxic effects may be synergistic, antagonistic, or additive, underscoring the need for more comprehensive research to accurately assess these complex interactions [44].

#### 3.7.2. Future Research Directions on the Impact of NPs on Reproductive Health

Future research on the impact of NPs on reproductive health should be expanded and intensified across several key areas. First, a more comprehensive understanding of the sources, distribution, and environmental fate of NPs across different matrices is urgently needed, coupled with accurate assessments of actual human exposure levels. Such data will facilitate more precise risk assessments concerning the impact of NPs on reproductive health [45].

Second, in-depth exploration of the molecular mechanisms by which NPs affect reproductive function, particularly their roles in regulating gene expression and modulating signal transduction pathways, will provide a theoretical foundation for developing targeted interventions. For instance, investigating how NPs influence the expression of key genes in germ cells and how these transcriptional alterations contribute to reproductive dysfunction represents a critical research priority [46].

Third, intensified research on the combined effects of NPs with other environmental pollutants is essential. Elucidating their joint toxic effects and underlying mechanisms is paramount for conducting holistic assessments of how environmental contaminants affect reproductive health. Concurrently, prioritizing human-based studies, including large-scale epidemiological investigations and well-designed clinical trials, to directly validate the effects of NPs on human reproductive health represents a vital future direction.

#### 3.7.3. Role of Policy and Regulation in Preventing and Controlling NPs-Induced Reproductive Damage

Policies and regulations play a pivotal role in the prevention and mitigation of NPs-induced reproductive damage. The establishment of stringent standards for plastic production and emissions, aimed at limiting the generation and release of NPs, can effectively reduce environmental contamination at the source. For example, policies restricting the use of single-use plastics and promoting the development and adoption of biodegradable alternatives, already implemented in several countries and regions, have contributed to reducing the environmental burden of NPs [47].

Strengthening the regulatory oversight of NP-related products, including the establishment of rigorous quality testing standards and certification systems, is necessary to ensure that plastic products on the market comply with safety requirements and to minimize consumer exposure risks. Concurrently, policies should incentivize research on the impact of NPs on reproductive health by increasing scientific funding, thereby providing a robust evidence base for the development of more effective prevention and mitigation strategies. Furthermore, policy-guided public education initiatives aimed at raising awareness of the potential hazards posed by NPs can promote public engagement in environmental protection, thereby collectively advancing efforts to prevent and mitigate NPs-induced reproductive damage.

## 4. Conclusions

The accumulating evidence underscores the significant potential of microplastics and nanoplastics (MPs/NPs) to adversely affect male reproductive health. Through mechanisms including oxidative stress, inflammation, endocrine disruption, and direct cellular damage, NP exposure has been unequivocally demonstrated in animal models to impair spermatogenesis, reduce sperm quality and motility, disrupt ovarian folliculogenesis, induce hormonal imbalances, and cause structural damage to reproductive tissues. Although pharmacological interventions and preventive strategies have shown promise in mitigating these effects in preclinical models, their translation to human applications requires rigorous validation through clinical studies.

Several critical challenges persist, including the precise quantification of human exposure levels, a comprehensive understanding of the combined effects of NPs with other environmental pollutants, and elucidation of the relevance of findings from model systems to human reproductive health. Moving forward, interdisciplinary research collaborations, robust epidemiological studies, and stringent regulatory measures will be essential to accurately assess risks and develop effective interventions. Addressing these knowledge gaps and implementing evidence-based policies is imperative to safeguard reproductive health globally in the face of escalating plastic pollution.

## Figures and Tables

**Table 1:** Presence of micro-nano plastics in human biological samples.

Types of MPs/NPs	MPs/NPs Size	Abundance of MPs/NPs	Specimen	References
PP, PET, PE	3 μm	(0.69 ± 0.84) particles/g (Based on tissue mass)	Lung tissue	[10]
PS, PVC, PET, PMMA, POM, PP	4~30 μm	3.2 particles/g (Based on tissue mass)	Liver tissue	[10]
PC, PA, PP	0.8~1.6 mm	(28.1 ± 15.4) particles/g (Based on tissue mass)	Colectomy	[10]
PE, PVC, PP	4~9 μm	-	Breastmilk	[10]
PU, PES, CPE	20~500 μm	39.5 particles/(10 mL)	Sputum	[10]
PP, PET, PS, PE, PA, PC, PVC, PU	20~800 μm	1~36 particles/g	Feces	[10]
PET, PE, PS	≥700 nm	1.6 g/mL	Blood	[11]
PVC, PP, PBS	20.34~307.29 μm	(2.70 ± 2.65) particles/g	Placenta	[12]

PP, polypropylene; PET, polyethylene terephthalate; PE, polyethylene; PVC, polyvinyl chloride; PBS, polybutylene succinate; PS, polystyrene; PC, polycarbonate; PA, polyamide; PU, polyurethane; PES, polyethersulfone; CPE, chlorinated polyethylene; PMMA, polymethyl methacrylate; POM, polyoxymethylene.

**Table 2:** Summary of Nanoplastic Reproductive Toxicity Studies.

Particle Type & Size	Experimental Model	Observed Reproductive Effects	Proposed Molecular Mechanisms	Reference
Polystyrene Nanoplastics (PS-NPs)	Mouse testicular tissue/germ cells	Accumulation in the testis, disruption of the blood-testis barrier, impaired spermatogenesis, and damage to spermatogenic cells.	Induces ferroptosis in spermatogenic cells. Nuclear factor erythroid 2-related factor 2 (Nrf2) plays a protective role in this process.	[33]
50 nm and 90 nm Polystyrene Nanoplastics (PS-NPs)	Mouse spermatocyte cell line (GC-2spd(ts))	Impaired cell proliferation and viability.	Disrupts cell cycle, autophagy, ferroptosis, and redox pathways; alters key metabolic pathways (e.g., amino acid, cyanoamino acid, purine/pyrimidine metabolism), suggesting metabolic reprogramming is involved.	[34]
50 nm and 90 nm Polystyrene Nanoplastics (PS-NPs)	Male Mice (in vivo)	Reduced sperm count and motility, increased sperm abnormality rate, and altered testosterone levels.	Disrupts the gut microbiota composition and alters the fecal metabolite profile. Suggests interference with male reproductive function via the “gut microbiota-metabolite” axis.	[35]
Polystyrene Nanoplastics (PS-NPs)	GC-2spd(ts)	Induction of ferroptosis, leading to male reproductive injury.	Promotes the degradation of ferroportin 1 (FPN1) by regulating its ubiquitination, leading to intracellular iron overload and lipid peroxidation.	[36]

## Data Availability

Data will be made available upon reasonable request.
